# Maximum likelihood-based estimation of diffusion coefficient is quick and reliable method for analyzing estradiol actions on surface receptor movements

**DOI:** 10.3389/fninf.2023.1005936

**Published:** 2023-03-08

**Authors:** Geza Makkai, Istvan M. Abraham, Klaudia Barabas, Soma Godo, David Ernszt, Tamas Kovacs, Gergely Kovacs, Szilard Szocs, Tibor Z. Janosi

**Affiliations:** ^1^Institute of Physiology, Medical School, University of Pécs, Pécs, Hungary; ^2^Nano-Bio-Imaging Core Facility at the Szentágothai Research Centre of the University of Pécs, Pécs, Hungary; ^3^Centre for Neuroscience, Szentágothai Research Centre, University of Pécs, Pécs, Hungary

**Keywords:** diffusion coefficient, maximum likelihood, mean square displacement, MLE, receptor movements

## Abstract

The rapid effects of estradiol on membrane receptors are in the focus of the estradiol research field, however, the molecular mechanisms of these non-classical estradiol actions are poorly understood. Since the lateral diffusion of membrane receptors is an important indicator of their function, a deeper understanding of the underlying mechanisms of non-classical estradiol actions can be achieved by investigating receptor dynamics. Diffusion coefficient is a crucial and widely used parameter to characterize the movement of receptors in the cell membrane. The aim of this study was to investigate the differences between maximum likelihood-based estimation (MLE) and mean square displacement (MSD) based calculation of diffusion coefficients. In this work we applied both MSD and MLE to calculate diffusion coefficients. Single particle trajectories were extracted from simulation as well as from α-amino-3-hydroxy-5-methyl-4-isoxazolepropionic acid (AMPA) receptor tracking in live estradiol-treated differentiated PC12 (dPC12) cells. The comparison of the obtained diffusion coefficients revealed the superiority of MLE over the generally used MSD analysis. Our results suggest the use of the MLE of diffusion coefficients because as it has a better performance, especially for large localization errors or slow receptor movements.

## Introduction

The diffusion coefficient is the most frequently defined parameter used to characterize receptor movements (De Keijzer et al., 2011; Knight and Falke, 2009; Knight et al., 2010; Matsuoka et al., 2009; Michalet and Berglund, 2012; Michalet, 2010; Pinaud and Dahan, 2011; Qian and Sheetz, 1991; Sahl et al., 2010; Schütz et al., 1997; Weigel et al., 2011).

The derivation of diffusion coefficient from mean square displacement (MSD) curve fitting (Matysik and Kraut, 2014) is a basic and frequently used method because it provides consistent results despite of the statistical shortcomings of MSD analysis (Saxton, 1997). The main problem with MSD analysis is that the overlapping time-averaging calculations in MSD curves from a single trajectory generate complex noise characteristics (Grebenkov, 2011; Qian and Sheetz, 1991). This resulted in an asymmetric distribution of the estimated diffusion constant around the true value that makes the interpretation of the results difficult (Yu, 2016). Another problem is that MSD cannot handle the uncertainty of the localization properly, in other words, the MSD requires the real coordinates of the particle to provide correct results. However, this is not the case in practice, because observed trajectories are compromised with both the localization error (Martin et al., 2002) and the motion blur effect (Savin and Doyle, 2005).

Maximum likelihood-based estimation (MLE) has already been successfully applied to estimate diffusion coefficients from single-particle tracking experiments (Shuang et al., 2013). The MLE is one of the most frequently used method in statistics to estimate arbitrary parameters of theoretical models describing the observed event by using recorded data. Changing the model’s parameters will alter the probability of the recorded dataset. MLE is an optimization method, that estimates a set of parameters that provides the maximal probability of the observed data. The MLE has asymptotically optimal properties, it determines the correct distribution of diffusion coefficients for a homogenous set of particles localized within a finite camera integration time and in the presence of localization error (Zacks, 1971). A comprehensive study on detailed comparison of MSD and MLE methods was recently published (Bullerjahn and Hummer, 2021), which concluded several advantages of the maximum likelihood estimator compared to other diffusion coefficient calculating methods.

There is a clear relation between the movement of cell surface receptors and their signal transduction activity. There are several single molecule detection (SMD) techniques to investigate this relationship. Events that result in clear changes, such as receptor ligand interactions can be studied by previously widely used analytical methods such as MSD curve analysis. However, for biological effects that cause only small variations in receptor movements but result in biologically significant changes, conventional methods can no longer be used for reliable investigation.

The reliability of the MSD and MLE methods were tested on simulated datasets as well as on data derived from live-cell experiments. For the live-cell measurements we detected changes in the surface movement of α-amino-3-hydroxy-5-methyl-4-isoxazolepropionic acid (AMPA) receptors after estradiol exposure.

The gonadal steroid 17β-estradiol (E2) is a powerful molecule playing a key role in learning and memory formation by influencing glutamatergic neurotransmission and synaptic plasticity (Kramár et al., 2009; Ledoux et al., 2009; Lu et al., 2019; Murakami et al., 2018; Teyler et al., 1980; Vierk et al., 2014; Wong and Moss, 1992). Besides its well-known classical actions, E2 can influence gene expression indirectly by rapidly altering the functions of membrane receptors and the activity of second messenger molecules. These are referred to as the non-classical effects of E2 (Rudolph et al., 2016). Although ample data have been accumulated on the rapid effects of E2 on learning and memory (Phan et al., 2015; Taxier et al., 2020), the molecular mechanisms are still largely unknown. Single-molecule tracking studies showed that the lateral diffusion of membrane receptors determine the activation state of membrane receptors and consequently the downstream signaling events (Kusumi et al., 2014).

The surface movement of glutamate receptors including AMPA receptors is pivotal in glutamatergic neurotransmission and synaptic plasticity (Babayan and Kramar, 2013; Penn et al., 2017).

Accordingly, measuring the diffusion parameters of the AMPARs can provide a better understanding of the non-classical E2 effects on learning and memory processes (Godó et al., 2021). Therefore, it is crucial to improve currently available methods to analyze membrane receptor movements.

Recent studies (Barabas et al., 2021; Godó et al., 2021) on lateral movement of receptors in the plasma membrane have demonstrated the value of the data extracted from SMD. SMD is a technique that can identify individual molecules and create the trajectories of these particles for detailed analysis. This allows deeper insights into the function of the receptors and helps us to understand the underlying mechanisms of different agents actions such as E2.

When examining the effect of E2 on the movement of AMPA receptors, because of the shortness of the detected trajectories and the larger localization error due to the specificity of the labeling, the MLE method has been proven to be more accurate in determining the diffusion coefficient of the AMPA receptors.

In this current manuscript we found that MLE method is better to analyze single molecule receptor movements by comparing the MSD and the MLE analysis of simulated and real, live-cell datasets.

## Materials and methods

### Simulated trajectories

A Matlab script was applied to generate sets of trajectories for two dimensional Brownian-diffusion with different characteristics. Besides the number of desired trajectories, the script allows the user to define the diffusion coefficient, the Gaussian localization error, the exposure time, the pixel size, the number of frames in each individual trajectory to customize the output according to the requirements. Moreover, there is an additional option that allows the user to turn the motion blur effect on or off.

### Measured trajectories

To collect trajectories of real immobilized and diffusing molecules we performed single-molecule imaging using total internal reflection fluorescence microscopy (TIRFM). Single-molecule imaging was carried out on an Olympus (Tokyo, Japan) IX81 fiber TIRF microscope equipped with Z-drift compensation (ZDC2) stage control, a plan apochromat objective (100X, NA 1.49, Olympus), and a humidified chamber heated to 37°C and containing 5% CO_2_. The dish containing dPC12 was mounted in the humidified chamber of the TIRF microscope immediately after *in vivo* labeling. A 491 nm diode laser (Olympus) was used to excite ATTO 488, and emission was detected above the 510 nm emission wavelength range. The angle of the excitation laser beam was set to reach a 100 nm penetration depth of the evanescent wave. A Hamamatsu 9100-13 electron-multiplying charge-coupled device (EMCCD) camera and Olympus Excellence Pro imaging software were used for image acquisition by TIRF microscopy. Image series were captured with 10-s sampling intervals and 33-ms acquisition times. Single-molecule tracking of labeled particles was performed with custom-made software written in C++ (WinATR, Kusumi Lab, Membrane Cooperativity Unit, OIST). The center of each particle was localized by two-dimensional Gaussian fitting, and the trajectory for each signal was created by a minimum step size linking algorithm that connected the localized dots in subsequent images. The trajectories were individually checked, and artifacts or tracks shorter than 15 frames were excluded from further analysis.

### Immobilized particles

To measure immobilized particles, we dried a droplet of ATTO 488-labeled antibodies directed against the extracellular N-terminal domain of rat GluR2 (1:1,000 in PBS, Alomone Labs) onto a glass bottomed dish. The dried dyes were covered with Prolong Gold Antifade Mountant (P10144, Thermo Fisher, Waltham, MA, USA). After 24 h, image series of immobilized ATTO-488 dyes were collected and analyzed as described above.

### AMPARs in live dPC12 cells

To detect GluR2-AMPAR molecules in the plasma membranes of differentiated PC12 (dPC12) (Godó et al., 2021), live-cell immunofluorescent labeling was performed. Before single-molecule imaging, dPC12 were incubated with ATTO 488-labeled antibodies directed against the extracellular N-terminal domain of rat GluR2 (1:100, Alomone Labs Cat #: AGC-005-AG) in dRPMI cell culture medium at 37°C for 6 min. During the measurement period of ATTO 488-GluR2-AMPAR, 20–30 image series were recorded. 17β-estradiol was applied immediately before imaging the dPC12 in dRPMI in 100 pM and 100 nM concentration dissolved in vehicle (EtOH).

### Calculation of diffusion coefficients

Mean square displacement curve (MSD) for each trajectory was calculated by the following equation (Matysik and Kraut, 2014; Yu, 2016):


$$\text{MSD}\,\left(m\,△\,T\right)=\frac{1}{N-m}\,\sum_{i=1}^{N-m}\left({\left(x_{i+m}-x_{i}\right)}^{2}+{\left(y_{i+m}-y_{i}\right)}^{2}\right)$$

where *x*_*i*_ and *y*_*i*_ are the observed coordinates of tracked particle, Δ*T*: time interval between two consecutive frames, *N*: total number of frames, and *m* as an independent variable represents the time delay (in frames) applied for the particular point of the MSD curve. The calculation of diffusion coefficients was implemented by three points linear fitting on the MSD curve. The parameters extracted from the MSD fitting are also provided by the Matlab script available in the Supplementary material.

In order to obtain the corresponding *D* value by MLE, the MLE was applied as previously described (Berglund, 2010). Δ*x*_*k*_ and Δ*y*_*k*_ represent the observed displacements (Δ*x*_*k*_ = *x*_*k* + 1_−*x*_*k*_ and Δ*y*_*k*_ = *y*_*k* + 1_−*y*_*k*_) arranged in *N*-component column vectors, where the total number of frames is equal to *N*+1. *x*_*n*_ and *y*_*n*_ are the coordinates of the signal’s center on the *n*th frame, as usual. The *N* × *N* covariance matrix (Σ) is defined by the following equation:


$$\Sigma_{i\,j}=\left\{ \begin{array}{c} 2\,D\,\Delta \,t-2\,\left(2\,D\,R\,\Delta \,t-\sigma^{2}\right),\mathrm{if}\,i=j\\ 2\,D\,R\,\Delta \,t-\sigma^{2},\mathrm{if}\,i=j\pm 1\\ 0,\text{otherwise} \end{array}\right.$$

where *D* is the diffusion coefficient, Δ*t* is frame integration time, σ is the static localization noise, *i* and *j* are the row and column indexes in the covariance matrix and *R* summarizes the motion blur effect.


$$R=\frac{1}{T}\,\int_{0}^{T}S\,\left(t\right)\,\left[1-S\,\left(t\right)\right]\,dt\,w\,h\,e\,r\,e\,S\,\left(t\right)=\int_{0}^{t}s\,\left(t^{\prime }\right)\,dt^{\prime }$$

where *s*(*t*) is the shutter function, in our case, *R* = 1/6 as a consequence of continuous illumination.

The likelihood was defined by the following function:


$$L\,\left(\Delta \,x,\Delta \,y\right)=-l\,o\,g\,\left|\Sigma \right|-\frac{1}{2}\,{\left(\Delta \,x\right)}^{T}\,\Sigma^{-1}\,\left(\Delta \,x\right)-\frac{1}{2}\,{\left(\Delta \,y\right)}^{T}\,\Sigma^{-1}\,\left(\Delta \,y\right)$$

The *D* and σ which provides the maximal likelihood is the estimated diffusion coefficient and static localization noise, respectively. The calculation of the determinant and the inverse of covariance matrix at each step of the optimization method can be a severe computational difficulty at high value of *N*. An approximation (Gray, 2005) based on the theory of circulant matrices is applicable (Berglund, 2010). In the script we defined a constant for the limit to switch between the direct and the simplified calculation method. Based on our experience we set the value of this constant to 1,001. When the number of frames exceeds 1,000 this simplified likelihood function is used for the global optimization, otherwise the direct likelihood function was applied. In this study the maximal length of trajectories was 1,000 frames, so the script applied the direct method for each trajectory. To estimate the area of molecule trajectories the convex hull for each trajectory was created by a Matlab script. Area of the molecule trajectory was defined as the area of this convex hull.

The Matlab script for the MLE based estimation of diffusion coefficient is available as a zip file available in the Supplementary material.

## Results

### Simulated trajectories

**FIGURE 1:**
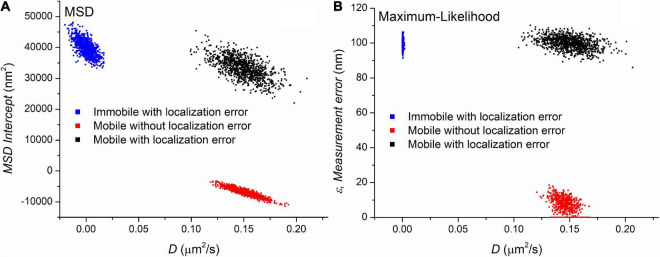
The parameters extracted by mean square displacement (MSD) **(A)** and maximum likelihood-based estimation (MLE) **(B)** based parameter estimation on three set of simulated trajectories. Each point on the graphs represents a set of parameters calculated from a trajectory. The value of diffusion coefficients is shown on the *x*-axis of both graphs. The *y*-axis represents another parameters provided by the diffusion coefficient’s estimation, namely they are the *y*-intercept of the linear fitting and the extracted localization error for the MSD and MLE graph, respectively. The number of trajectories is 1,000 in each group.

Three sets of trajectories were generated with MSD and MLE estimations assuming the presence of the blur effect due to continuous recording. Each set containing 1,000 trajectories with a length of 501 frames differed in the values of the diffusion coefficient and the localization error. The first group contained immobile (*D* = 0μ*m*^2^/*s*) trajectories in the presence of ε=100*nm* localization uncertainty. The second set contained mobile (*D* = 0.15μ*m*^2^/*s*) trajectories without any localization error (ε=0*nm*). The last group simulated trajectories recorded on moving particles (*D* = 0.15μ*m*^2^/*s*) with ε=100*nm* measurement error. Figure 1 shows the parameters provided by the MSD and MLE.

Figure 1 demonstrates that both methods clearly separate the distinct sets of trajectories. The MLE reliably provides the expected parameters while diffusion coefficients provided by the MSD method are in good agreement with the theoretical values. A minor difference between the two methods is observed between the distribution of diffusion coefficients from the mobile trajectories with no localization error. The MLE estimates the diffusion coefficients with less standard deviation (SD). However, this observation has no significance in the single molecule imaging because the lack of localization error is a purely theoretical category. The main difference between the two sets of data is the distribution of diffusion coefficients extracted from the immobile trajectories. While the MSD based diffusion coefficients show some variability around the group’s average of 0 μm^2^/s, the distribution of the same parameter in the same group provided by the MLE is much narrower. Since this scenario can easily happen if we observe slow particles, this finding has a great importance, and we went further to investigate it in detail.

To investigate this phenomenon, another set of trajectories were created and analyzed. While the localization error was constant (ε=100*nm*), both the length of trajectories and the diffusion coefficients were altered. The length was altered from 11 to 1,001 frames. The diffusion coefficients had the following values: 0.01μ*m*^2^/*s*, 0.02μ*m*^2^/*s*, 0.05μ*m*^2^/*s*, 0.1μ*m*^2^/*s*, 0.2μ*m*^2^/*s*, and 0.5μ*m*^2^/*s*. The number of randomly created trajectories in each group was 1,000. The set of raw simulated data is available in the Supplementary material.

The group means provide satisfactory estimation of the diffusion coefficient when the number of steps (i.e., the number of frames minus one) is equal or above 20. At the shortest trajectories (length is equal to 10 steps) some uncertainty is present independently of the applied method. In this case the mean values slightly differ from the expected ones. This finding confirms the legitimacy of the general practice that in studies with single-molecule tracking the trajectories below the length of 15 steps are omitted from further analysis.

**FIGURE 2:**
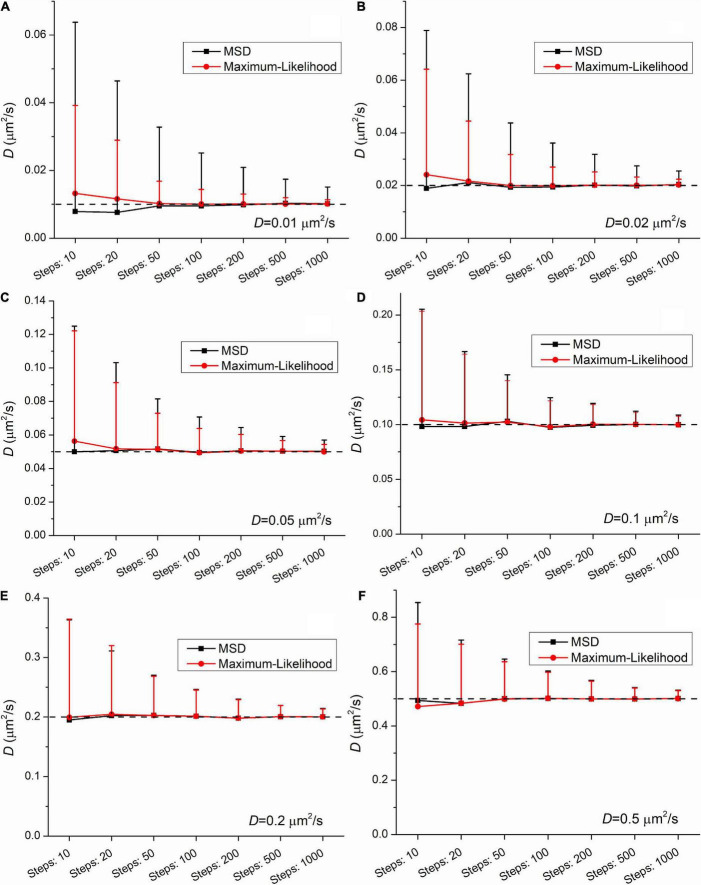
Mean and standard deviation (SD) values of diffusion coefficients extracted from a set of trajectories (*N* = 1,000) simulated with the following diffusion coefficients: **(A)** 0.01μ*m*^2^/*s*, **(B)** 0.02μ*m*^2^/*s*, **(C)** 0.05μ*m*^2^/*s*, **(D)** 0.1μ*m*^2^/*s*, **(E)** 0.2μ*m*^2^/*s*, **(F)** 0.5μ*m*^2^/*s* as a function of the length of trajectories. The diffusion coefficients were extracted by both the mean square displacement (MSD) (black) and maximum likelihood-based estimation (MLE) (red) method.

**FIGURE 3:**
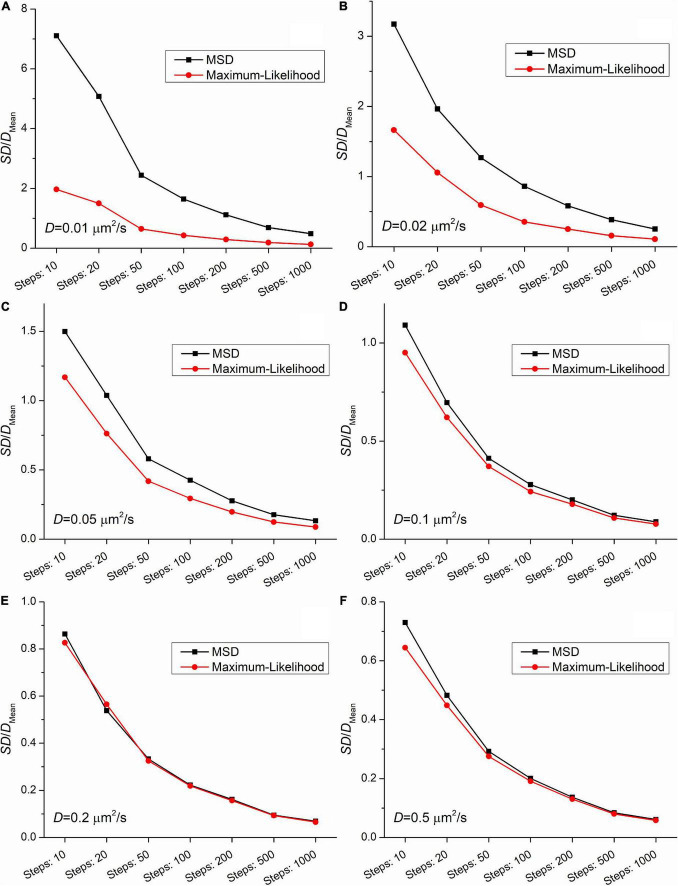
The coefficients of variation (the ratio of the SD and the mean from Figure 2) as a function of the length of trajectories. The diffusion coefficients for the simulation were: **(A)** 0.01μ*m*^2^/*s*, **(B)** 0.02μ*m*^2^/*s*, **(C)** 0.05μ*m*^2^/*s*, **(D)** 0.1μ*m*^2^/*s*, **(E)** 0.2μ*m*^2^/*s*, **(F)** 0.5μ*m*^2^/*s*.

Figures 2, 3 demonstrate that the SD and coefficient of variation (CoV) of diffusion coefficients derived by MSD are larger than the corresponding values extracted by MLE. In the two slowest group of trajectories (*D* = 0.01μ*m*^2^/*s* and *D* = 0.02μ*m*^2^/*s*) both the CoV and SD parameters provided by the two analyses differ to a large extent and this difference is independent of the trajectory length. The values of CoV of the MSD based diffusion coefficients for the slowest trajectories (*D* = 0.01μ*m*^2^/*s*) are approximately three times higher than the corresponding values extracted by the MLE. In the case of the slightly faster group (*D* = 0.02μ*m*^2^/*s*) the application of the MSD method provides two times higher CoV values for the diffusion coefficients than the MLE based analysis. In the group simulated with *D* = 0.05μ*m*^2^/*s* the MSD provided values of CoV for the diffusion coefficients exceed the same values from MLE based calculation by 30%. This difference between the values of SD and CoV diminish slowly with the increasing diffusion coefficient. The values of SD and CoV are crucial in several types of statistical test, and a broader distribution can easily disguise a slight but a real difference between the investigated groups. While the provided mean values calculated by the MLE as well as the MSD method are in good agreement with the expected values, the distribution of the group’s diffusions coefficients are narrower in each set of trajectories proving a better performance of MLE based calculation on simulated data.

### Measured immobile particles

To test the usability of MLE on measured trajectories we carried out an analysis on trajectories recorded on immobile particles at different temperatures. However, the investigated particles are named “immobile” some movement is always present. For these particles diffusion coefficients are approximately two orders of magnitude smaller than receptor’s diffusion coefficients. We expected more intense movement at elevated temperature. The trajectories are available in the Supplementary material.

**FIGURE 4:**
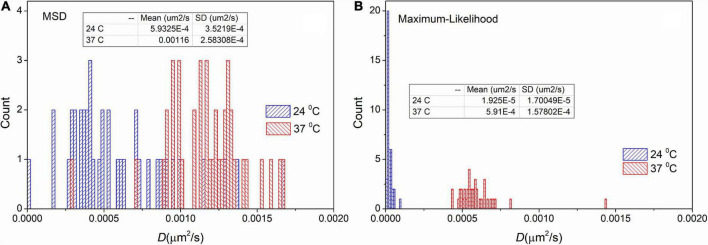
Distribution of diffusion coefficients derived from trajectories recorded on immobile particles. The measurement was carried out on different temperatures and the extracted trajectories were analyzed by the mean square displacement (MSD) **(A)** and the maximum likelihood-based estimation (MLE) **(B)** method. The inserted table shows the mean and SD values for each group, respectively.

Figure 4 shows the distribution of diffusion coefficients measured at different temperatures on immobile samples. These distributions confirm the result derived from the simulated data. There is a shift in the mean values 5.9⋅10^−4^μ*m*^2^/*s* and 3.0⋅10^−5^μ*m*^2^/*s* for the trajectories measured at 24°C. As it was expected the mean values are higher (1.2⋅10^−3^μ*m*^2^/*s* and 6.1⋅10^−4^μ*m*^2^/*s*) at 37°C. More importantly, the values of SD are significantly decreased by applying the MLE. While provided values of SD by the MSD method are 3.5⋅10^−4^μ*m*^2^/*s* and 2.6⋅10^−4^μ*m*^2^/*s*, the distributions from MLE based analysis are significantly narrower (the corresponding SD values are: 2.7⋅10^−5^μ*m*^2^/*s* and 1.7⋅10^−4^μ*m*^2^/*s*). These findings match the results of our previous *in silico* experiments.

### Trajectories measured on live dPC12 cells

Analysis performed on simulated data and immobile particles showed that the MLE had remarkable performance which occasionally exceeded the abilities of MSD based method. To compare the two approaches also in live-cell experiments, we tested their usability and reliability in an experimental model that has been routinely used in our laboratory. Therefore, comprehensive analysis was carried out on AMPA receptor (GluR2-AMPAR) trajectories measured in live dPC12 cells after E2 or vehicle treatment.

**FIGURE 5:**
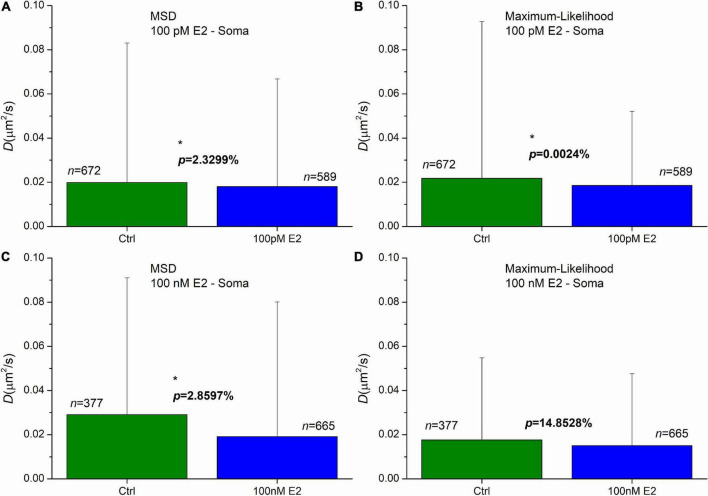
The effect of E2 treatment on the diffusion coefficient of GluR2-AMPAR molecules in the soma’s plasma membranes of dPC12, live-cell. The E2 treatments were carried out by the concentration of 100 pM **(A,B)** and 100 nM **(C,D)**. Both the mean square displacement (MSD) **(A,C)** and the maximum likelihood-based estimation (MLE) **(B,D)** methods were used for further analysis to obtain the diffusion coefficients from the recorded trajectories. The graphs represent the groups as mean and SD values. The probability values of significant differences calculated by Kolmogorov–Smirnov test (*p < 0.05) and the number of trajectories in each group are also shown.

Administration of 100 pM E2 induced a significant decrease of diffusion coefficients in AMPAR in soma in the first 20 min after the treatment. The means were decreased to 0.018 μ*m*^2^/*s* and 0.019 μ*m*^2^/*s*, while the control’s mean values were 0.020 μ*m*^2^/*s* and 0.022 μ*m*^2^/*s* for the MSD and MLE, respectively (Figures 5A, B). The probability of significance was *p* = 2.33% and less than 0.01% for the MSD and MLE method, respectively. The application of 100 nM E2 highlighted the difference between the two calculation methods. While analysis conducted by the MLE (Figure 5D) showed no effect (*p* = 14.85%) after E2 administration, the MSD method provided a significant decrease of the diffusion coefficients (Figure 5C). In this case the mean of diffusion coefficients was 0.019 μ*m*^2^/*s*, which was significantly lower (probability of significance is *p* = 2.86%) than the same value in the control group 0.029 μ*m*^2^/*s*.

The result of MLE can be surprising as the lower E2 concentration (100 pM) evoked a significant decrease of the diffusion coefficients, while the administration of the higher dose of E2 (100 nM) did not induce any change. This effect was previously investigated (Godó et al., 2021) and it was revealed that the difference may be the consequence of GPER1 internalization in the soma induced by 100 nM E2. It was also demonstrated that both ERβ and GPER1 are required for the effect of E2. The higher dose of E2 induced elimination of GPER1 preventing E2 to cause decrease of the diffusion coefficient.

In soma, the 100 nM E2 treatment has distinct effect, based on the two calculation methods. On one hand, the MLE does not reveal any significant effect due to E2 treatment, on the other hand the application of E2 significantly decreases the diffusion coefficients based on statistics on the MSD results. Previous study (Godó et al., 2021) has shown that GPER1 internalization depletes the GPER1 which is crucial for the effectiveness of E2 in soma, indicating the propriety of MLE based result.

**FIGURE 6:**
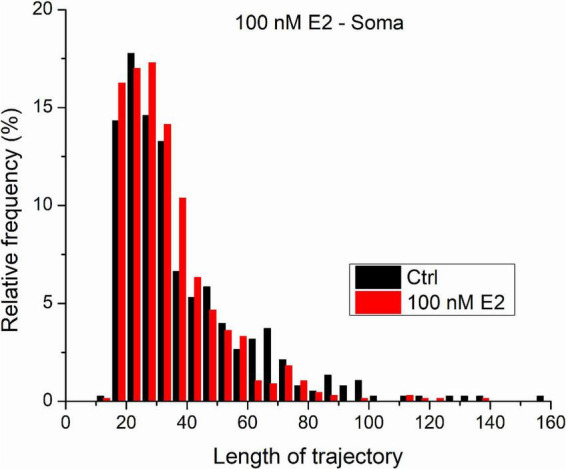
The trajectories length distribution from GluR2-AMPAR molecules in the soma’s plasma membranes of dPC12, live-cell in control state and after administration of 100 nM E2.

Figure 6 shows the distribution length distribution of trajectories measured on GluR2-AMPAR molecules in the somatic plasma membrane of living dPC12 cells both in control state and after the administration of 100 nM E2. The vast majority of trajectories are shorter than 50 steps. Our previous results on simulated trajectories proved that MLE provides more reliable result on trajectories characterized with similar parameters (*D* = 0.02μ*m*^2^/*s* and the length are less or equal to 100 steps). Based on this we think that in this case we can acknowledge the MLE provided results and statistical statement.

## Discussion

The focus of the current study was to examine in depth the differences between MLE and MSD-based methods. First, we used simulated trajectories, which are suitable to detect localization errors. Our results show that while the obtained group averages of the diffusion coefficients perfectly corresponded to the expected values regardless of the computational methods, the SD values of the diffusion coefficients were significantly lower for the *D* = 0μ*m*^2^/*s* (immobile trajectories with localization error) group using the MLE method. This difference between the distribution of the diffusion coefficient values is the consequence of the fundamental difference between the two methods. On one hand the MSD based calculation does not constrain the sign of the diffusion coefficient, therefore the *D* values, especially for slow or immobile trajectories, often have a negative sign, which is difficult to interpret. On the other hand, the MLE method does not provide sub-zero diffusion coefficients, so the distribution of *D* values is much narrower.

Secondly, the reliability of the methods was investigated, also using simulated trajectories to compare mean and SD values for low diffusion coefficients. The length of the trajectories and expected diffusion coefficients characterized the randomly generated trajectories in these groups. The analysis of the set of simulated trajectories showed no difference between the two methods in terms of mean values. Both analyses provided good estimates of the expected values. These results were consistent with our previous finding, namely that the MLE method gave more accurate estimation of diffusion coefficients. The SD value of diffusion coefficients from MSD method exceeded the SD provided by MLE based calculation when the value of *D* was less than 0.2μ*m*^2^/*s*. In addition, both mean and SD values were identical when the diffusion coefficient was greater or equal to 0.2μ*m*^2^/*s*. The analysis following numerical simulation showed that the MLE outperforms the MSD as a data analysis tool.

Regarding measured immobile trajectories at different temperatures, the two methods provided similar values for the average of the diffusion coefficient in any analyzed groups. According to the expectations, the higher temperature evoked a more intense movement, which was reflected in increased diffusion coefficients. The experiment clearly confirmed that the distribution of diffusion coefficients provided by the MLE is much narrower than the distribution calculated by the MSD approach. The reason for this difference is the following: in contrast to MLE method MSD is less effective in separating the static localization noise from the diffusion generated displacement, which causes increased uncertainty in the calculated diffusion coefficients. This phenomenon is pronounced when the localization error exceeds the expected displacement by diffusion (i.e., in the case of so-called immobile particles).

Finally, the two methods were tested on trajectories collected from live dPC12 cells. The effect of E2 on the movement of GluR2-AMPAR molecules was investigated in somata of dPC12 cells. On the one hand, the 100 pM E2 treatment significantly decreased the mean value of diffusion coefficients by applying either the MSD or the MLE method. On the other hand, the two calculation methods resulted in conflicting results when comparing the effect of 100 nM E2 in the soma. The MSD method showed a significant alteration in the diffusion coefficients of GluR2-AMPAR molecules, while the MLE demonstrated no effect. The result of MLE is consistent with the previously reported ineffectiveness of 100 nM E2 in the soma, due to GPER1 internalization. The investigation of length distribution of the trajectories and the results gained from simulated trajectories reveals that for this set of trajectories the MLE provides more reliable diffusion coefficients. So, the statistical result extracted from MLE based calculation seems to be more reliable and accurate in this particular case.

## Conclusion

The performed analysis conducted on simulated trajectories revealed that the provided mean values of diffusion coefficients are in good agreement with the theoretical values, regardless of the applied method. The superiority of MLE based calculation over MSD was shown by examination of the coefficients of variation (ratio of SD and the mean) for the distribution of the estimated diffusion coefficients. The CoV is remarkably lower by using MLE based method instead of the application of MSD based in the case of slow particle movement.

The results of simulation were confirmed by the results extracted from immobile trajectories measured at different temperatures. The distribution of diffusion coefficients is undoubtedly narrower in the case of MLE making the interpretation of obtained results easier.

Moreover, our findings were tested on AMPA receptor trajectories measured in live dPC12 cells after estradiol-treatment. The two calculation methods provided conflicting results when comparing the effect of 100 nM E2 in the soma.

On the one hand, MSD is less reliable for short trajectories or trajectories characterized with small diffusion coefficients. Moreover, MSD does not effectively separate the localization error from diffusion. On the other hand, MLE is applicable on short and slow trajectories, and it does separate the localization error from the movement. The superiority of the MLE method was demonstrated on simulated as well as on measured trajectories in live cells.

These results indicate that MLE method is one of the first recommended approach to analyze data obtained in single-molecule imaging measurements.

## Data availability statement

The original contributions presented in this study are included in the article/Supplementary material, further inquiries can be directed to the corresponding author.

## Author contributions

IA, KB, and TJ contributed to conception and design of the study. DE, TK, and SG were involved in sample preparation for the TIRF measurements. SG performed the TIRF measurements. KB, SG, and GK extracted trajectories from measured videos. TJ created the Matlab script for analyzing trajectories. SS and GM checked and optimized the script. GM and TJ performed the statistical analysis and wrote the first draft of the manuscript. KB, DE, TK, GK, and SG wrote sections of the manuscript. All authors contributed to manuscript revision, read, and approved the submitted version.
