# Supplementary figures and images for: Maximum likelihood-based estimation of diffusion coefficient is quick and reliable method for analyzing estradiol actions on surface receptor movements
Source: Front Neuroinform. 2023 Mar 8;17:1005936. doi: 10.3389/fninf.2023.1005936 (PMC10031098; doi:10.3389/fninf.2023.1005936)

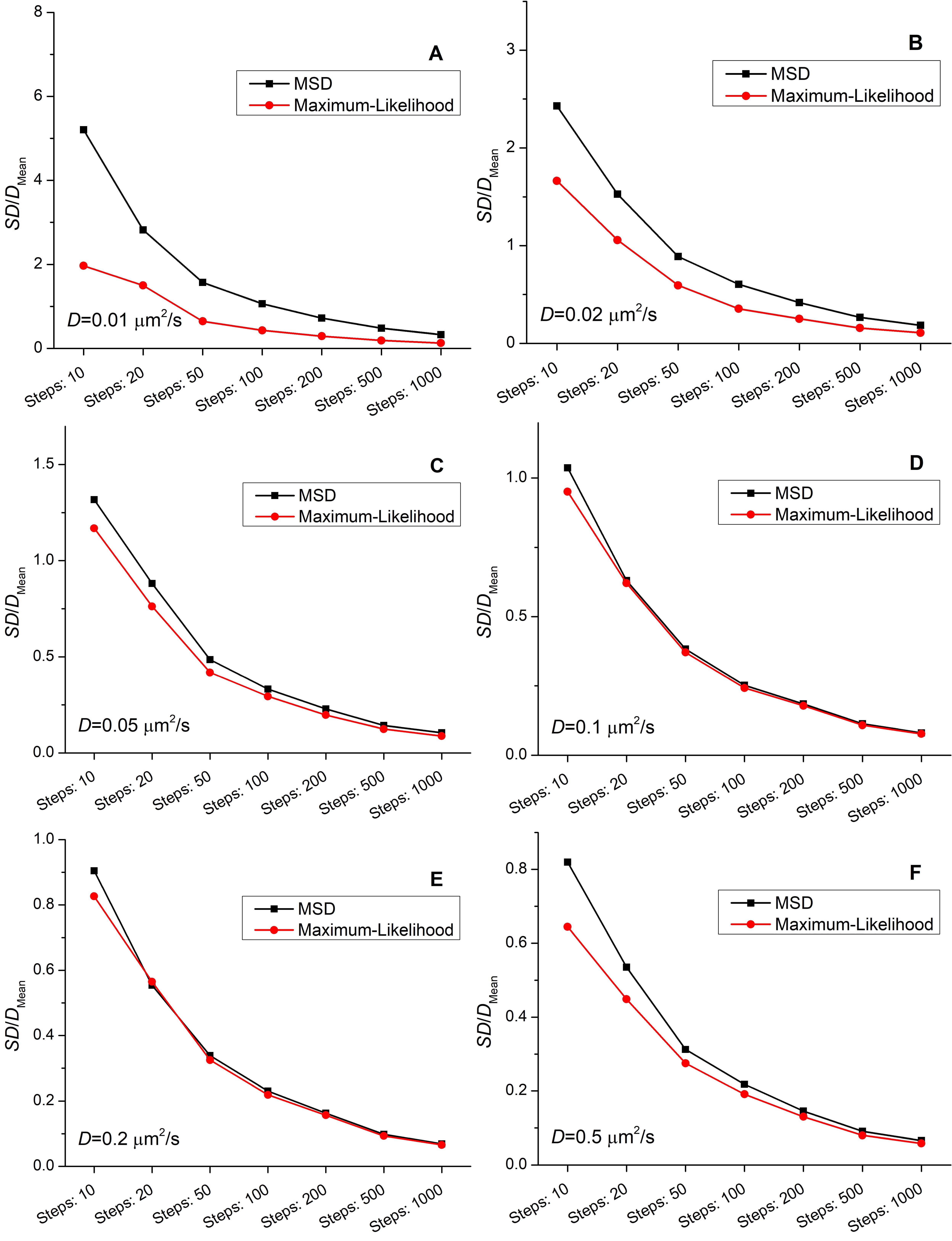

Supplement: Supplementary file 10 [file Data_Sheet_10.ZIP › Revision_Figures/CoV/4Points_CoV.jpg]

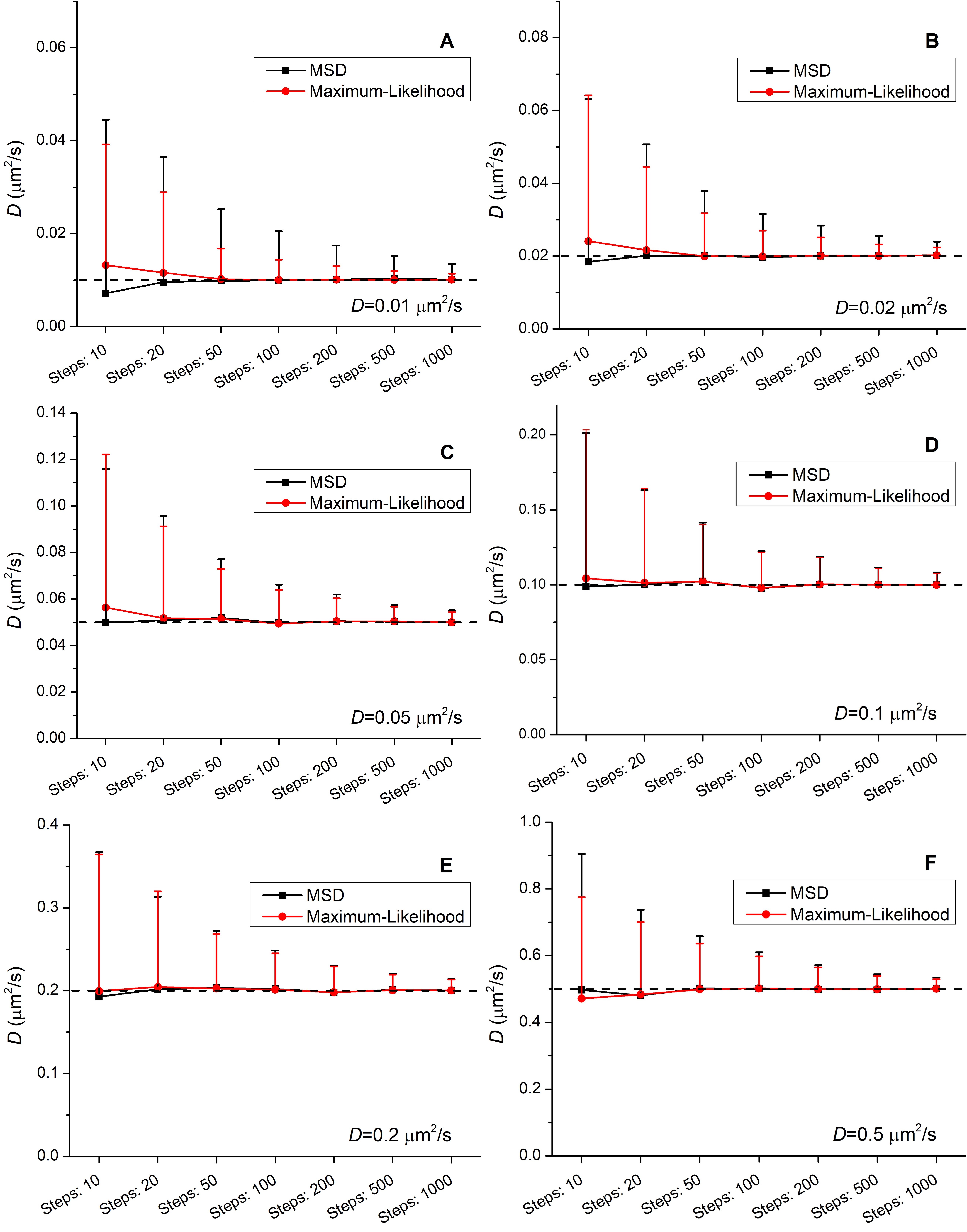

Supplement: Supplementary file 10 [file Data_Sheet_10.ZIP › Revision_Figures/CoV/4Points_SD.jpg]

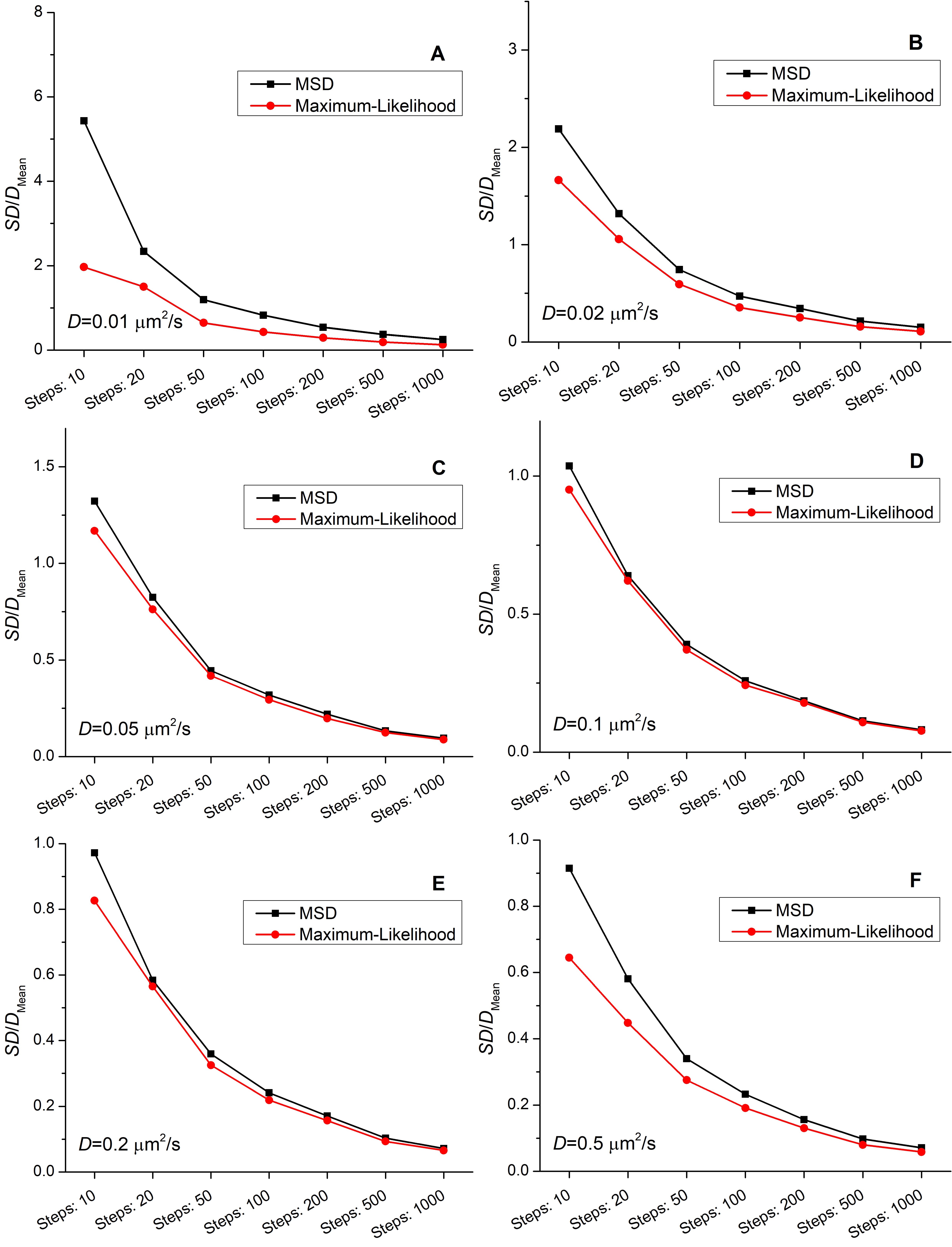

Supplement: Supplementary file 10 [file Data_Sheet_10.ZIP › Revision_Figures/CoV/5Points_CoV.jpg]

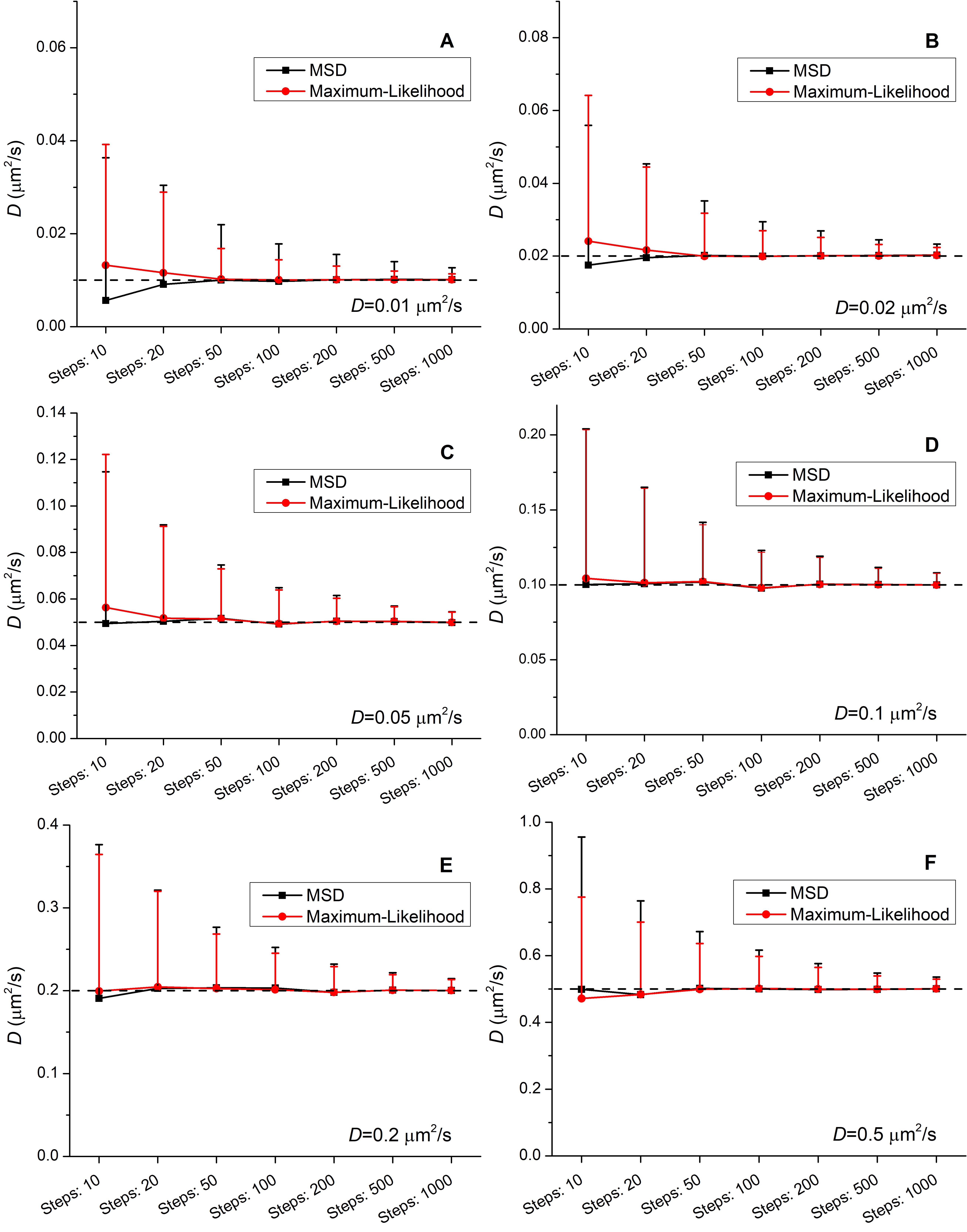

Supplement: Supplementary file 10 [file Data_Sheet_10.ZIP › Revision_Figures/CoV/5Points_SD.jpg]

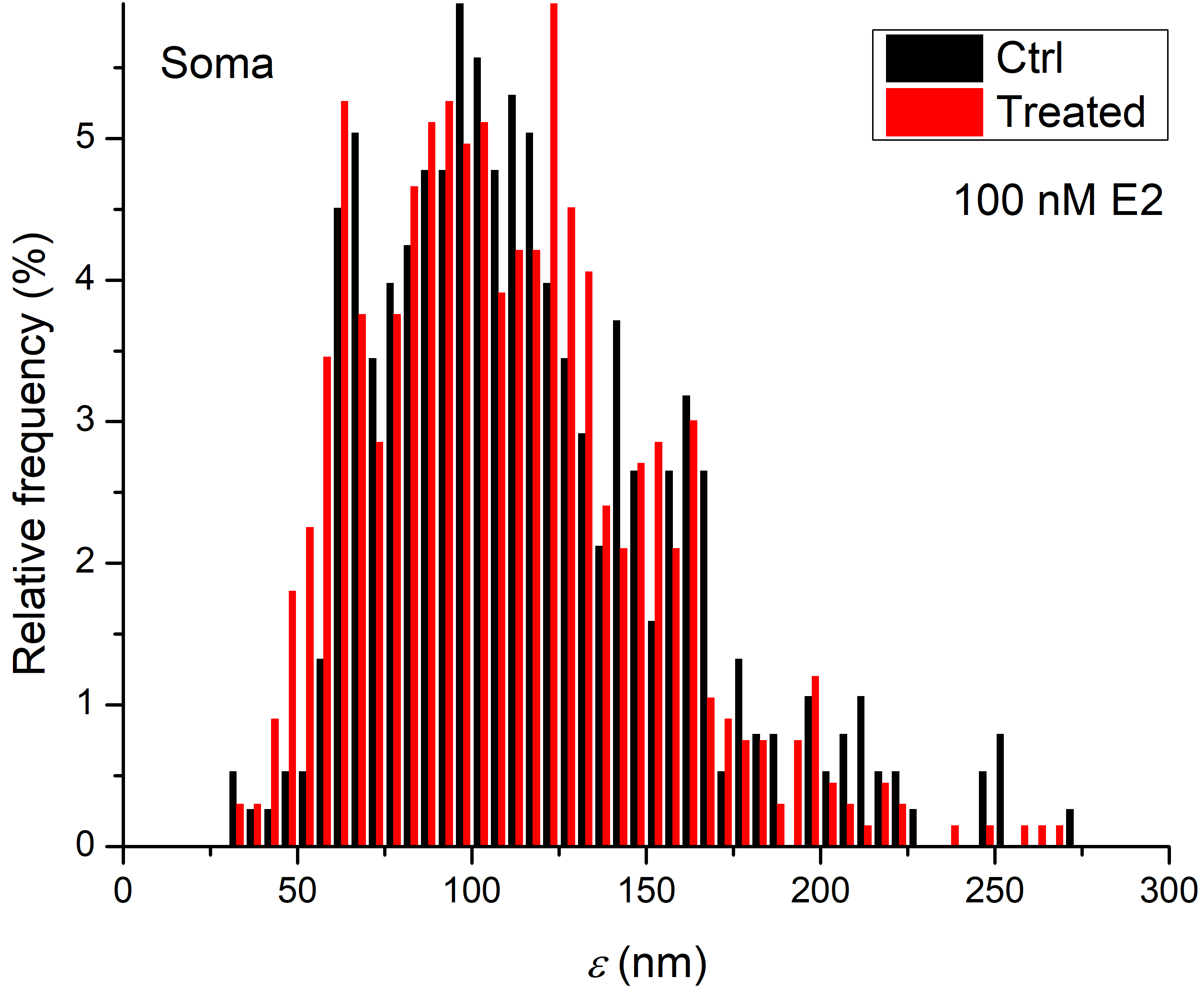

Supplement: Supplementary file 10 [file Data_Sheet_10.ZIP › Revision_Figures/LocalizationError/LocalizationError_Soma_100nM.png]

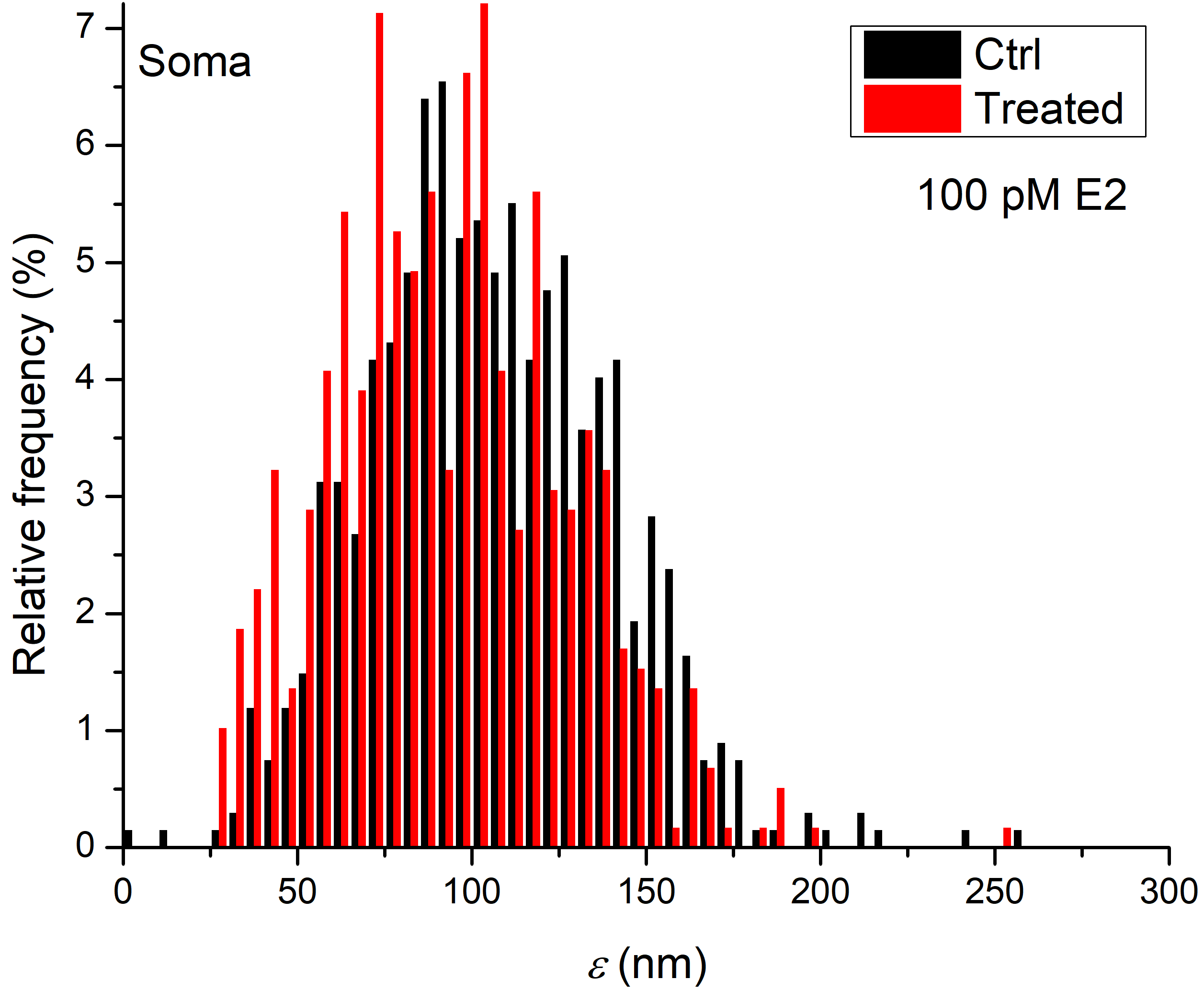

Supplement: Supplementary file 10 [file Data_Sheet_10.ZIP › Revision_Figures/LocalizationError/LocalizationError_Soma_100pM.png]
